# Are *Babesia vogeli* genotypes associated with *Rhipicephalus sanguineus* and *Rhipicephalus linnaei* distribution?

**DOI:** 10.1186/s13071-025-06923-8

**Published:** 2025-07-13

**Authors:** Vinícius Baggio-Souza, Laura Berger, Rafaela Mallmann-Bohn, Adeyldes Oliveira Reis, Lay Greco Basilio, Yasmin Santanna Pereira, Luiza Tirelli, Renata Fagundes-Moreira, João Vitor dos Santos Alves da Silva, Lorena Freitas das Neves, Ludmila Rodrigues Moroz, Huarrisson Azevedo Santos, Maria E. Marecos Espinoza, Cíntia Daudt, Fabio Gregori, Aline Giroto-Soares, Flávio Roberto Chaves da Silva, João Luis Garcia, Daniel Moura de Aguiar, Maristela Peckle Peixoto, José Manuel Venzal, Filipe Dantas-Torres, Marcos Rogério André, João Fabio Soares

**Affiliations:** 1 Laboratory of Protozoology and Vector-Borne Rickettsioses - ProtozooVet, Faculty of Veterinary – FAVET, Federal University of Rio Grande do Sul - UFRGS, Porto Alegre, Rio Grande do Sul Brazil; 2 Department of Public Health, Experimental and Forensic Medicine, University of Pavia, Pavia, Italy; 3 Vector-Borne Bioagents Laboratory (VBBL), Department of Pathology, Reproduction and One Health, School of Agricultural and Veterinary Sciences, São Paulo State University (FCAV/UNESP), Jaboticabal, São Paulo Brazil; 4 Department of Pathology and Clinics, Federal University of Bahia, Salvador, Bahia Brazil; 5 Department of Epidemiology and Public Health, Veterinary Institute, Federal Rural University of Rio de Janeiro (UFRRJ), Seropédica, Rio de Janeiro, Brazil; 6 Department of Veterinary Clinics, National University of Asunción, Asunción, Paraguay; 7 Virology and Parasitology Laboratory, Veterinary Medicine Program, Federal University of Acre, Rio Branco, Acre Brazil; 8 School of Veterinary Medicine and Animal Science, University of São Paulo, São Paulo, SP Brazil; 9 Department of Preventive Veterinary Medicine, State University of Londrina, Londrina, Paraná Brazil; 10 Virology and Rickettsioses Laboratory of the Veterinary Hospital, Faculty of Veterinary Medicine, Federal University of Mato Grosso, Cuiabá, Mato Grosso Brazil; 11 Department of Animal Parasitology, Veterinary Institute, Federal Rural University of Rio de Janeiro (UFRRJ), Seropédica, Rio de Janeiro, Brazil; 12 Vector and Transmitted Diseases Laboratory, CENUR Litoral Norte – Salto, Universidad de la República, Rivera, Salto, Uruguay; 13 Aggeu Magalhães Institute, Oswaldo Cruz Foundation (Fiocruz), Recife, Pernambuco Brazil

**Keywords:** Canine babesiosis, Tick-borne diseases, Brown dog ticks

## Abstract

**Background:**

In South America, *Babesia vogeli* is the primary causative agent of canine babesiosis, and brown dog ticks (*Rhipicephalus sanguineus* sensu lato) are the vectors. The recent separation of brown dog ticks into *Rhipicephalus sanguineus* sensu stricto (“temperate lineage”) and *Rhipicephalus linnaei* (“tropical lineage”) raised suspicions of the possibility of two distinct *Babesia* genotypes or even species being transmitted by these tick species.

**Methods:**

To investigate this hypothesis, dog blood samples from Brazil (eight states), Paraguay, and Uruguay were collected to determine the genetic diversity of *B. vogeli* in South America. The samples were collected from temperate regions (southern Brazil and Uruguay), where the putative vector is *R. sanguineus*, and from the tropical areas (southeastern, midwestern, northeastern, and northern Brazil and Paraguay), where *R. linnaei* is the vector. DNA samples from *B. vogeli*-positive dogs were extracted to amplify the 18S ribosomal RNA, internal transcribed spacers 1 and 2, heat shock protein 70, cytochrome c oxidase subunit 1, cytochrome oxidase c subunit 3, and cytochrome b genes. The sequences obtained were aligned with available *B. vogeli* sequences in GenBank and other homologous sequences to construct phylogenetic trees, haplotype networks, and matrices.

**Results:**

Our haplotypic and phylogenetic analyses congruently indicated the existence of one genotype in temperate areas and another in tropical areas, where *R. sanguineus* and *R. linnaei* act as vectors, respectively. While the percentage of similarity varied among the evaluated genetic markers, the results indicated a clear differentiation between the *B. vogeli* genotypes associated with temperate and tropical regions.

**Conclusions:**

Our data indicate the existence of two *B. vogeli* genotypes in South America, associated with temperate and tropical areas. This contributes to a better understanding of *B. vogeli’s* genetic diversity and opens new avenues for researching the ecology and coevolution of *B. vogeli* genotypes and their tick vectors. Owing to their correlation with the climatic region and the historical nomenclature of their vectors, we suggest the nomenclature of “temperate” and “tropical” *B. vogeli* genotypes.

**Graphical Abstract:**

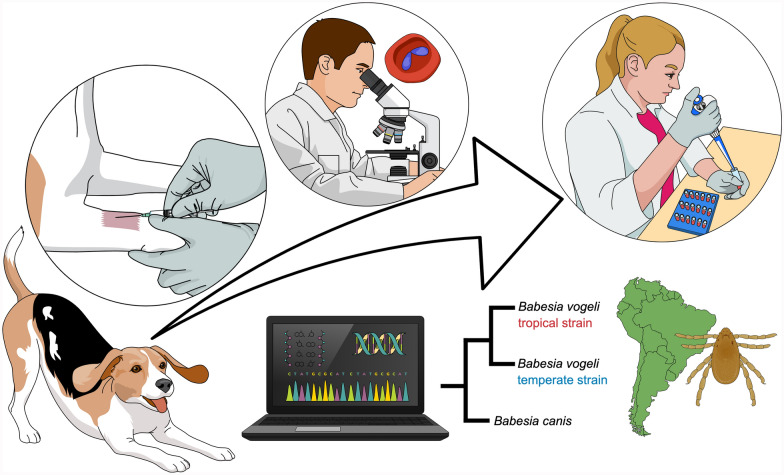

**Supplementary Information:**

The online version contains supplementary material available at 10.1186/s13071-025-06923-8.

## Background

Among the three causative agents of canine piroplasmosis in South America, namely *Rangelia vitalii* [1], *Babesia gibsoni* [2], and *Babesia vogeli* [3], the latter has the most extensive geographical distribution. *Babesia vogeli* is a blood parasite transmitted by ixodid ticks [4] that holds significant veterinary importance across the Americas, Europe, Africa, Asia, and Oceania, where it causes canine babesiosis, a disease that primarily affects juvenile and immunocompromised animals [5]. The most frequent clinical sign is mild anemia. However, severe systemic illness characterized by fever, hemolytic anemia, thrombocytopenia, lethargy, anorexia, icterus, and hemoglobinuria may occur in immunocompromised or juvenile dogs, underscoring its impact on canine health in endemic regions [6].

*Babesia vogeli* is primarily transmitted by brown dog ticks (*Rhipicephalus sanguineus* sensu lato). Biological, morphological, and phylogenetic evidence gathered during the past 20 years indicate that ticks historically identified as '*Rhipicephalus sanguineus'* in temperate and tropical regions comprised two distinct species, initially designated as *R. sanguineus* “temperate” and “tropical” lineages [7]. These species were later formally recognized as *Rhipicephalus sanguineus* sensu stricto (referred to from now on as *R. sanguineus*) [8], the former “temperate” strain, and *Rhipicephalus linnaei* [9], the “tropical” strain. It is now recognized that *R. sanguineus* is present in temperate regions of the Americas, Europe, and Asia, whereas *R. linnaei* is found in the tropical areas of the Americas, Africa, Asia, and Australia, with some rare regions where both species coexist in sympatry.

This taxonomic revision has significant implications for research and disease control, as vector competence varies between these species. For example, the prevalence of *Ehrlichia canis* in dogs is generally higher in areas where *R. linnaei* is present than in those where *R. sanguineus* is present [10], suggesting that their role as vectors of *E. canis* and possibly other pathogens may vary.

The molecular characterization of *B. vogeli* genotypes and their vectors in South America may help understand the transmission dynamics, epidemiology, and clinical significance of canine babesiosis. Therefore, this study aimed to investigate the genetic diversity of *B. vogeli* in infected dogs across different Brazilian regions, Uruguay, and Paraguay, and to correlate it with the geographical distribution of the vector tick populations.

## Methods

### Samples and DNA extraction

Conveniently collected ethylenediaminetetraacetic acid (EDTA)-blood samples from dogs positive for *B. vogeli* from different Brazilian states—Acre (*n* = 2), Mato Grosso (*n* = 9), Distrito Federal (*n* = 3), Bahia (*n* = 16), São Paulo (*n* = 7), Rio de Janeiro (*n* = 9), Paraná (*n* = 8), and Rio Grande do Sul (*n* = 155)—as well as from Paraguay (*n* = 30) and Uruguay (*n* = 13), were included in this study. The samples were primarily sourced from partner universities, originating from surplus diagnostic material previously stored in their institutional sample banks, with the requirement that all represented autochthonous cases tested positive by polymerase chain reaction (PCR) or blood smear. In addition, the Laboratory of Protozoology and Vector-borne Rickettsioses at the Federal University of Rio Grande do Sul prospectively received samples from the state of Rio Grande do Sul during this study for diagnostic purposes. Samples were subjected to DNA extraction using the PureLink® Genomic DNA Mini Kit (Invitrogen, Carlsbad, CA, USA). DNA purity and concentration were assessed at 260/280 nm using a NanoDrop spectrophotometer (Thermo Scientific, USA), and the extracted DNA samples were stored at –20 °C until further use.

### PCR assays

A primer set (forward: Bvog751 and reverse: Bvog926) was designed in this study to differentiate *B. vogeli* and *R. vitalii* infections. The GenBank accession numbers of the sequences used to design the primers were AB248733, EF527401, and AB248732 for *B. vogeli* and JF279603 for *R. vitalii*. This primer amplifies a fragment of approximately 150 base pairs from the heat shock protein 70 gene (*hsp70*) (Table 1). The specificity of the PCR assay was determined by testing genomic DNA samples of each of the following agents: Apicomplexan protozoa: *B. vogeli*, *Babesia bovis*, *Babesia bigemina*, *Babesia caballi*, *Theileria equi*, *Cytauxzoon* sp., *Hepatozoon canis*, *Toxoplasma gondii*, and *Neospora caninum*; Kinetoplastida protozoa: *Leishmania infantum* and *Trypanosoma cruzi*; bacteria: *Rickettsia rickettsii*, *Rickettsia bellii*, *Ehrlichia canis*, and *Coxiella* spp. (origin of the samples is available at [11]). To test *hsp70* primer sensitivity, three aliquots of a sequenced *B. vogeli* sample were prepared at concentrations of 30 ng/μl, 3 ng/μl, and 1 ng/μl to determine the minimum detectable DNA concentration required for successful amplification.

**Table 1:** Genetic targets, primers, expected size (in base pairs), and thermal cycling protocols used in this study. References for each primer and protocol are also included

Target	Primers (5′–3′)	Size	Protocol	Reference
18S rRNA	Piro 0F (GCC AGT AGT CAT ATG CTT GTC TCA)	~1500	Denaturation at 95 °C for 5 min; 35 cycles at 94 °C for 1 min 30 s, 58 °C for 1 min, and 72 °C for 2 min; and extension at 72 °C for 10 min	Kawabuchi et al. [47], Soares et al. [1], Mongruel et al. [48]
	Piro 6R (CTC CTT CCT YTA AGT GAT AAG GTT CAC)			
	Piro 5.5R^a^ (CCT YTA AGT GAT AAG GTT CAC AAA ACT T)			
	Bab 2f^a^ (CCG TGC TAA TTG TAG GGC TAA TAC A)			
	Bab 2f2^a^ (CTT TGA GAA ATT AGA GTG TTT)			
	Bab 2r^a^ (TTC CTT AGA TGT GGT AGC CGT TTC)			
ITS1	F (GCT GCG TCC TTC ATC GTT GTG)	~300	Denaturation at 94 °C for 2 min; 30 cycles at 94 °C for 30 s, 60–66 °C for 30 s, and 72 °C for 1 min 30 s; and extension at 72 °C for 7 min	Bostrom et al. [49]
	R (CGA TCG AGT GAT CCG GTG AAT TA)			
ITS2	F (GTG AAC CTT ATC ACT TAA AGG)	~300	Denaturation at 96 °C for 3 min; 30 cycles at 94 °C for 30 s, 56 °C for 30 s, and 72 °C for 1 min 30 s; extension at 72 °C for 7 min	Duarte et al. [50]
	R (CAA CTC CTC CAC GCA ATC G)			
*hsp70*	Bvog751F^b^ (CCG TAT CCC CAA GAT CCA GC)	~170	Denaturation at 95 °C for 5 min; 30 cycles of 95 °C for 30 s, 60 °C for 30 s, and 72 °C for 30 s; and final extension of 72 °C for 5 min	This study
	Bvog926R^b^ (AGA GGG GAG CAA CGT CCA A)			
	F1 (CAT GAA GCA CTG GCC HTT CAA)	~740	Denaturation at 95 °C for 5 min; 36 cycles at 95 °C for 15 s, 58.5–60 °C for 30 s, and 72 °C for 1 min 30 s; and extension at 72 °C for 7 min	Soares et al. [1]
	R1^a^ (GCN CKG CTG ATG GTG GTG TTG TA)			
	F2^a^ (GGA TCA ACA AYG GMA AGA AC)	~720		
	R2 (GBA GGT TGT TGT CCT TVG TCA T)			
*cox1*	F (GGA AGT GGW ACW GGW TGG AC)	~900	Denaturation at 95 °C for 1 min; 35 cycles at 95 °C for 15 s, 45–57 °C for 30 s, and 72 °C for 1 min; and extension at 72 °C for 10 min	Schreeg et al. [42]
	R (TTC GGT ATT GCA TGC CTT G)			
*cox3*	F (ACT GTC AGC TAA AAC GTA TC)	~700	Denaturation at 94 °C for 5 min; 40 cycles at 94 °C for 20 s, 55 °C for 30 s, and 68 °C for 1 min 30 s; and extension at 72 °C for 7 min	Schreeg et al. [42]
	R (ACA GGA TTA GAT ACC CTG G)			
*cytb*	F (TTA GTG AAG GAA CTT GAC AGG T)	~900	Denaturation at 95 °C for 5 s; 35 cycles at 94 °C for 20 s, 58 °C for 30 s, and 72 °C for 1 min 30 s; and extension at 72 °C for 7 min	Schreeg et al. [42]
	R (CGG TTA ATC TTT CCT ATT CCT TAC G)			

^a^Internal primers used for sequencing

^b^Primers used on the screening PCR

Samples negative in the screening PCR (Table 1, Primers Bvog751F and Bvog926R) were removed from the study. Positive samples were then amplified for the designated genes via PCR using 0.26 µl of Platinum Taq DNA Polymerase (Invitrogen, Carlsbad, CA, USA), 1.5 mM of magnesium chloride (Invitrogen, Carlsbad, CA, USA), 0.2 µM of each dNTP (Invitrogen, Carlsbad, CA, USA), 10X PCR buffer (100 mM Tris–HCl, pH 9.0, 500 mM KCl), 1 µl of each forward and reverse primers, 2 µl of template DNA, and sterile ultra-pure water to complete a 25 µl reaction volume. Amplicons were visualized on a 1.5% agarose gel, using 5 µl of amplified sample and 0.5 µl of Blue Green Loading Dye I (Nova Biotecnologia, Cotia, Brazil) under light-emitting diode (LED) transillumination. PCR-positive samples were purified using the PureLink PCR Purification Kit (Thermo Fisher Scientific, Waltham, MA, USA) according to the manufacturer’s protocol. ACTGene Molecular Analyses Ltd. performed DNA sequencing (Rio Grande do Sul, Brazil).

All other primers and thermal cycling protocols used in this study were based on previously published data and are described in Table 1.

### Phylogenetic and genetic diversity analyses

The sequence quality was evaluated using PHPH, a web-based tool for electropherogram quality analysis (http://lbi.cenargen.embrapa.br/phph/), and the expected length of each fragment was determined accordingly. Those that did not meet the minimum requirements were excluded from the study. Forward and reverse sequences were aligned and trimmed (error probability = 0.05) to generate consensus sequences using BioEdit version 7.2.5 (North Carolina State University). The consensus sequences were compared with those deposited in GenBank using Standard Nucleotide BLAST (https://blast.ncbi.nlm.nih.gov/Blast.cgi). The obtained sequences were aligned with corresponding GenBank homologous sequences via MAFFT version 7 [12] and trimmed (error probability = 0.05) in MEGA11 [13]. The most appropriate nucleotide substitution model was determined via the Bayesian information criterion (BIC) in IQ-Trees [14, 15] (http://iqtree.cibiv.univie.ac.at/). Bayesian analyses were performed via MrBayes version 3.7 through the CIPRES online service [16], with the Markov chain Monte Carlo (MCMC) algorithm with two independent runs of 100 million generations each and a 25% burn-in. The resulting trees were visualized and edited in FigTree version 1.4.4. An alignment with *B. vogeli* and *Babesia canis* was used to construct a haplotype network analysis with PopArt version 1.7. Pairwise distance matrices were generated in MEGA11 with *B. vogeli* sequences from regions where *R. sanguineus* and *R. linnaei* are vectors and homologous sequences.

Alignments with the samples obtained in this study and sequences from *B. vogeli* and *B. canis* retrieved from GenBank were created for the 18S rRNA, *cytb*, *cox1*, and *cox3* genes. These alignments served as the basis for genetic diversity analysis, which was carried out via DnaSP version 6.12.03 software to compute nucleotide diversity, haplotype diversity, number of haplotypes, and average number of nucleotide differences. The TCS network was also applied through population analysis with reticulate trees (popART) software to construct the haplotype network.

## Results

**Fig. 1:**
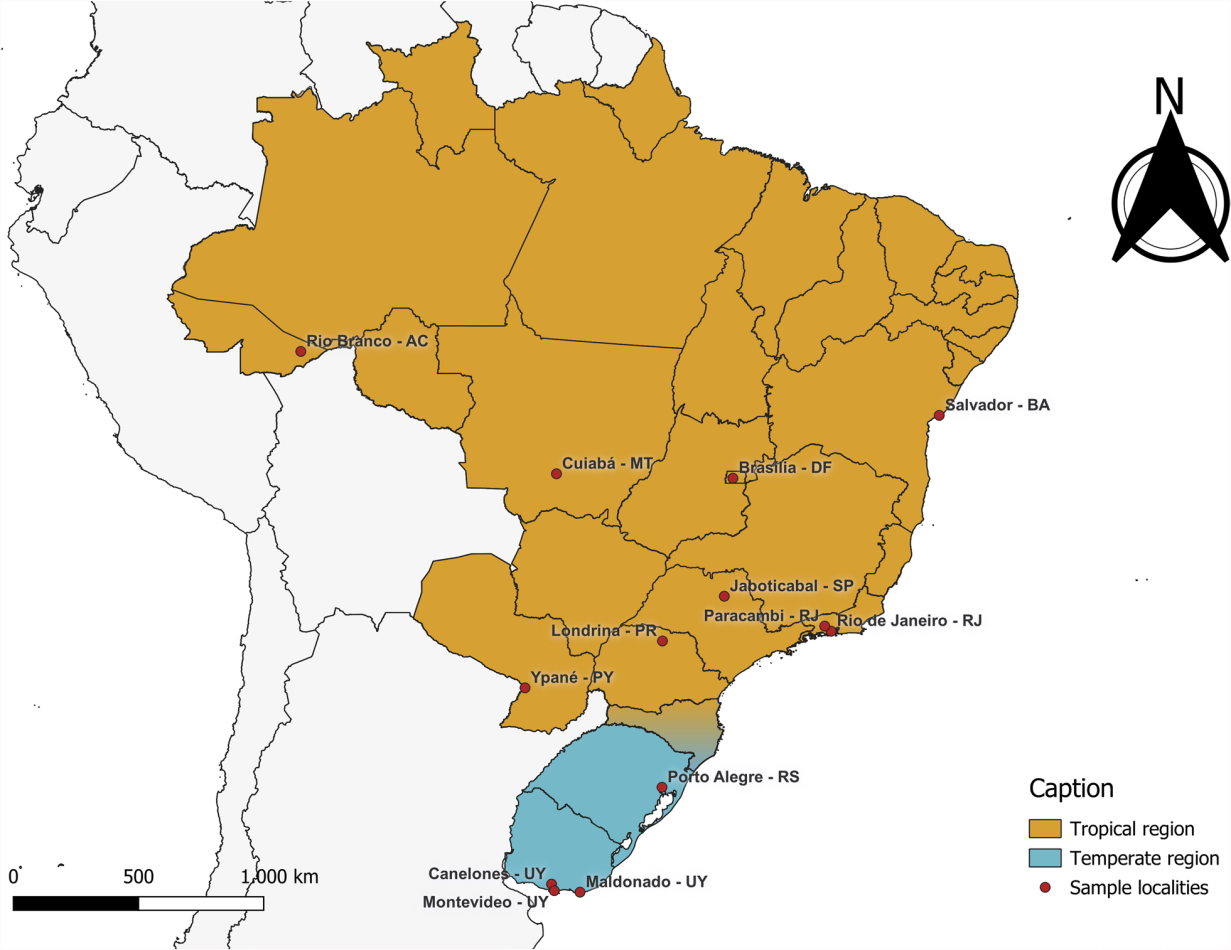
Map of South America showing sample locations (red dots). Tropical regions are colored in orange and temperate regions in blue

A map showing the origin of the sequences obtained in this study is available in Fig. 1. Sequences from regions where *R. sanguineus* is the probable vector differed across all evaluated genes compared with those from *R. linnaei*-endemic regions. Despite prior screening for *B.* *vogeli*, some samples from Brazilian states failed to amplify or yield readable sequences and were thus excluded from further analyses.

In total, 252 blood DNA samples obtained from dogs with suspected *B. vogeli* infection were subjected to PCR analyses. As most of the *B. vogeli* sequences deposited in GenBank are from tropical regions, only samples from the temperate region were amplified and sequenced for the 18S rRNA gene. A total of 24 samples (eight from the temperate region and 16 from the tropical region) that previously amplified in the screening primer were selected for sequencing the respective set of genes, resulting in 4/5 sequences for the 18S rRNA, 10/24 for ITS1, 15/24 for ITS2, 13/24 for *cox1*, 18/24 for *cox3*, 12/24 for *cytb*, and 11/24 for *hsp70*. One 18S rRNA sequence from Rio Grande do Sul and three from Uruguay were excluded because of their poor quality.

The similarity between *B. vogeli* sequences from temperate regions and sequences from GenBank was 98.7% for the ITS1, 99.8–100% for the ITS2, 99.3% for the *cox1* gene, 99.5% for *cox3*, 99.8% for *hsp70*, and 99.9% for 18S rRNA. One 18S rRNA sequence exhibited 100% identity to a US GenBank deposit, albeit with 99% query coverage. All sequences from *R. linnaei*-endemic regions presented 100% similarity and query coverage to those of *B. vogeli* in GenBank.

Phylogenetic analyses revealed distinct clustering of *B. vogeli* genotypes on the basis of the 18S rRNA, ITS1, ITS2, *hsp70*, *cox1*, *cox3*, and *cytb*. *Babesia vogeli* genotypes were designated “temperate” or “tropical” to reflect their association with *R. sanguineus* or *R. linnaei* vectors, respectively, on the basis of geographic origin.

**Fig. 2:**
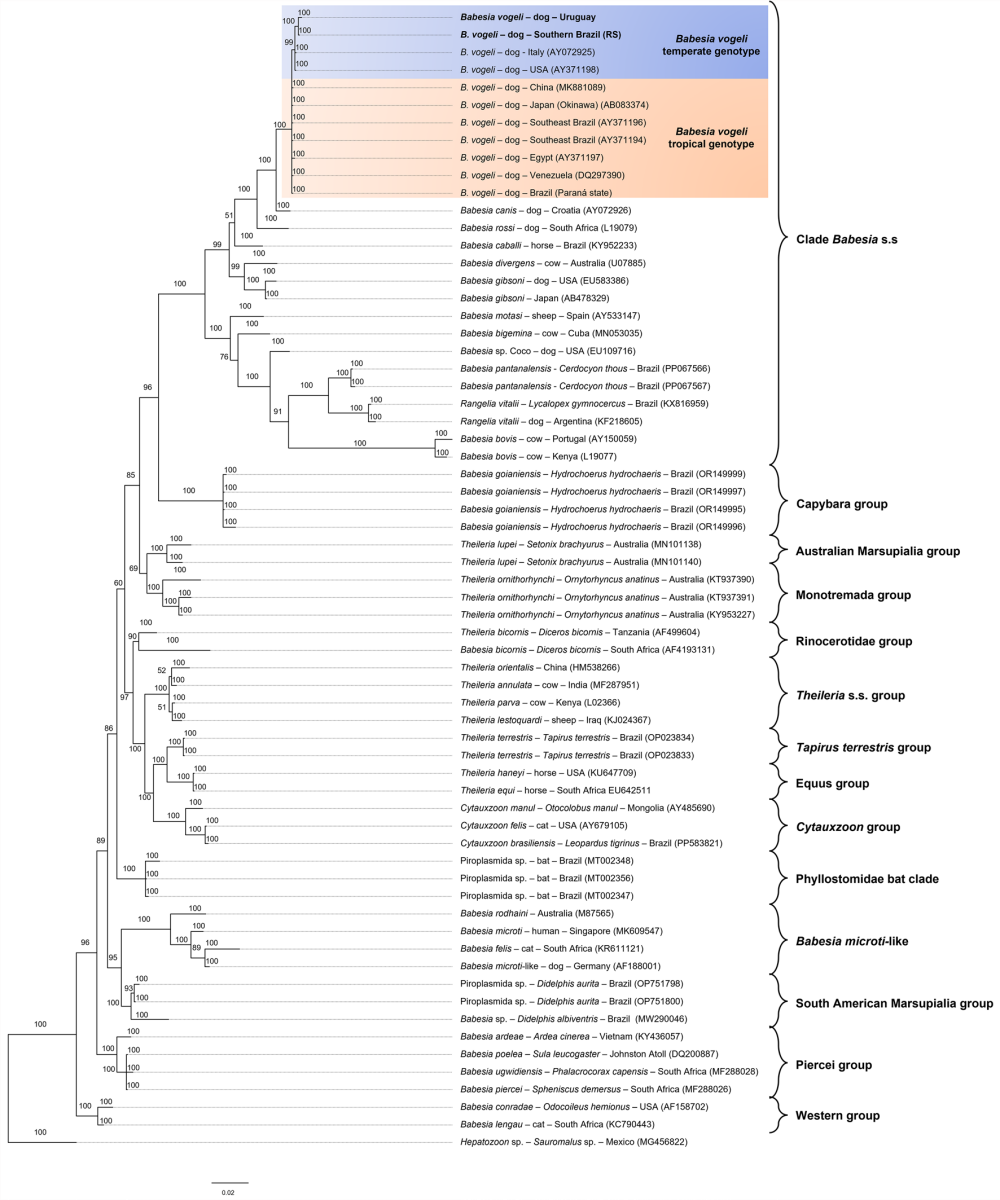
Bayesian phylogenetic tree based on the 18S rRNA gene constructed from a final alignment of 1014 informative sites. The substitution model (TN + F + I + G4) was determined via the IQ-Tree web server (available at: http://iqtree.cibiv.univie.ac.at/), with rate parameters of A–G (1.8745) and C–T (3.9615) (invariable sites, 0.516; gamma, 0.3757). The consensus tree was generated via MrBayes version 3.2.7. Sequences from temperate regions are highlighted with a blue background, whereas those from tropical regions are highlighted with an orange background. The sequences from this study are indicated in bold. Trees were generated with 100 million iterations and a 25% burn-in rate. *Hepatozoon* sp. (MG456822) was used as an outgroup

The 18S rRNA-based phylogenetic analyses used 500, 800, and > 1000 base pairs. Only analyses with 800 or more base pairs resolved distinct genotypes (Fig. 2). The final alignment included four samples from temperate regions (two from this study and two from GenBank) and seven from tropical regions (one from this study and six from GenBank). The genetic divergence among the 18S rRNA sequences ranged from 0.2% to 0.4%. In the 18S rRNA-based divergence matrix, *B. canis* showed genetic divergence values of 2.8% and 2.5% compared with *B. vogeli* “temperate” and “tropical genotypes.” *Babesia rossi* showed genetic divergence values of 5.4% and 5% when compared with *B. vogeli* “temperate” and “tropical genotypes,” respectively (Additional file 1).

The results of the haplotype analyses (Table 2) revealed two main haplotypes across all the studied loci (*cox1*, 18S rRNA, and *cytb*). While mitochondrial genes presented higher nucleotide diversity values (*Pi* = 0.00575 for *cox1* and 0.00449 for *cytb*) than the conserved 18S rRNA (*Pi* = 0.00098), these values remained relatively low. The positive Tajima’s D (*cox1*, 1.38; *cytb*, 1.32) and high Fu’s Fs values (*cox1*, 6.27; *cytb*, 5.13) suggest population subdivision, a pattern consistent with restricted gene flow between genotypes associated with the distinct vectors, namely *R. sanguineus* (putative vector in temperate regions) and *R. linnaei* (tropical regions).

**Table 2:** Results of haplotype analyses for *cox1*, 18S rRNA, and *cytb*. Analysis generated in DnaSP for Windows version 6.12.03

Parameter	*cox1*	18S rRNA	*cytb*
No. of variable sites	8	3	6
Total no. of mutations	8	3	6
Nucleotide diversity	0.00575	0.00098	0.00449
No. of haplotypes	2	2	2
Haplotype diversity	0.467	0.409	0.467
Tajima’s D	1.3814 (*P* > 0.1; ns)	0.77220 (*P* > 0.1; ns)	1.3246 (*P* > 0.1; ns)
Fu’s Fs	6.273	2.895	5.130

*ns* not significant

**Fig. 3:**
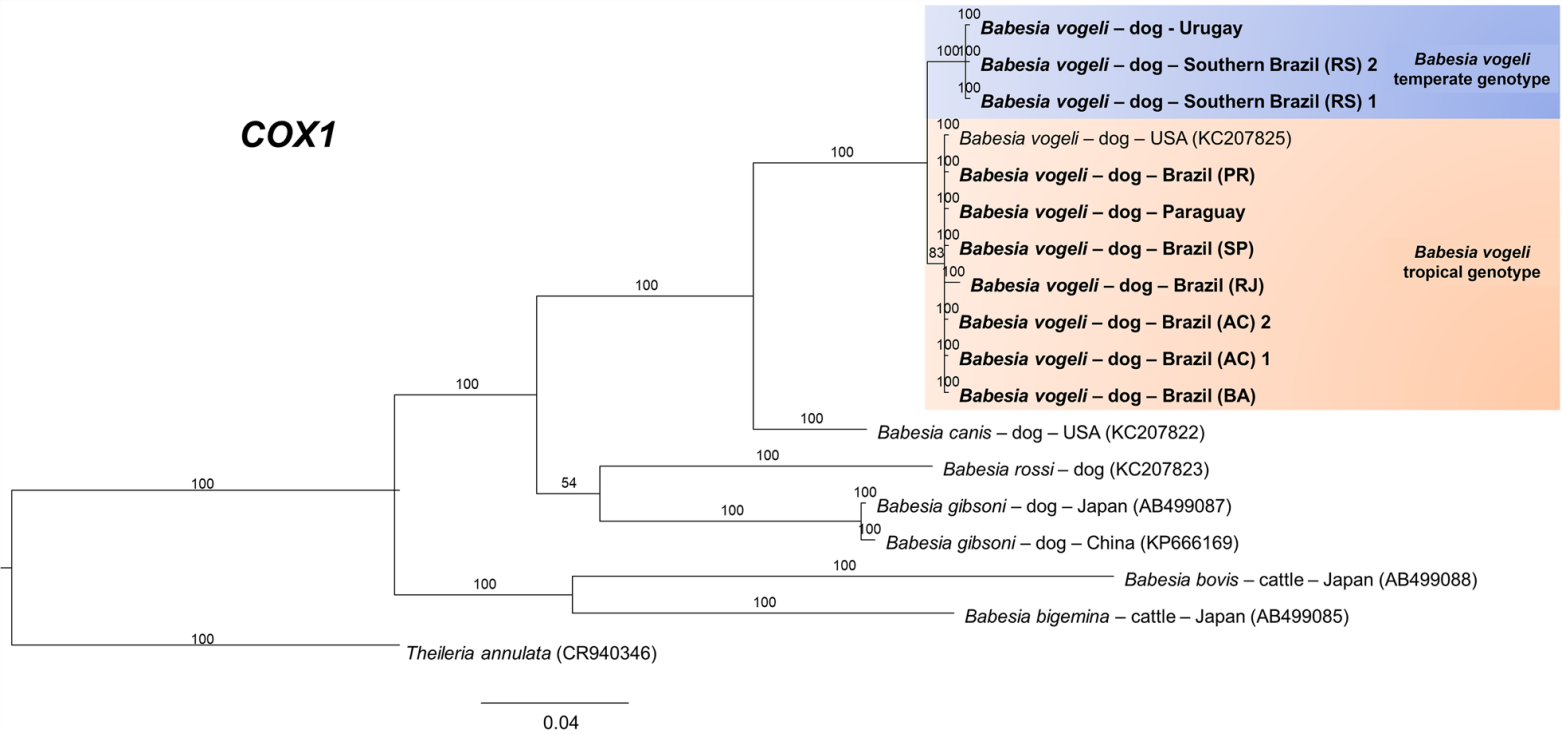
Bayesian phylogenetic tree for the *cox1* gene constructed from a final alignment of 19 sequences with 652 informative sites. The substitution model (K3Pu + F + G4) was determined via the IQ-Tree web server (available at: http://iqtree.cibiv.univie.ac.at/), with rate parameters of A–G (14.8019), A–T (9.4397), C–G (9.4397), and C–G (14.8019). The consensus tree was generated via MrBayes version 3.2.7. Sequences from temperate regions are highlighted with a blue background, whereas those from tropical regions are highlighted with an orange background. The sequences from this study are indicated in bold. Trees were generated with 100 million iterations and a 25% burn-in rate. *Theileria annulata* (CR940346) was used as an outgroup

**Fig. 4:**
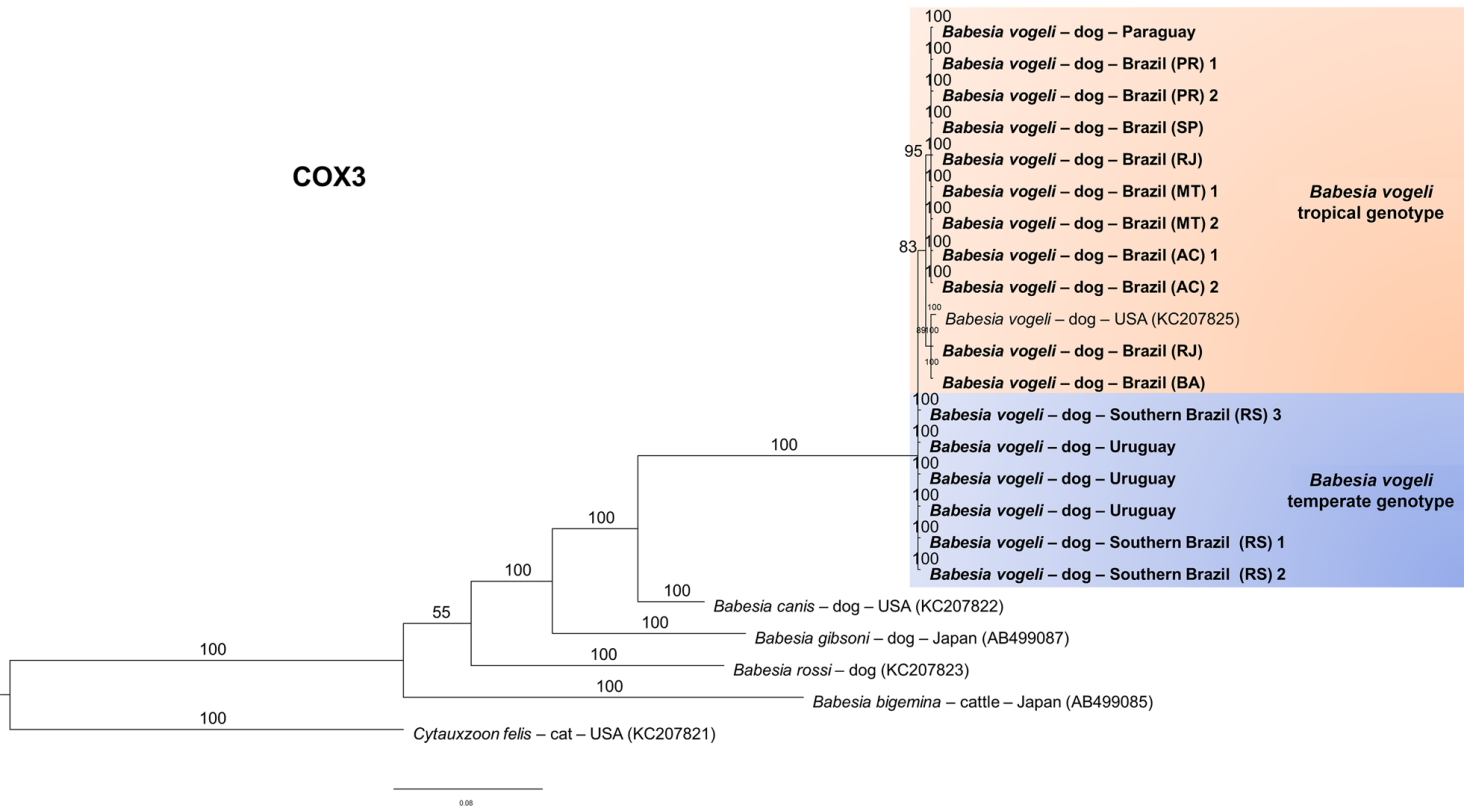
Bayesian phylogenetic tree based on the *cox3* gene constructed from a final alignment of 641 informative sites. The substitution model (K3Pu + F + G4) was determined via the IQ-Tree web server (available at: http://iqtree.cibiv.univie.ac.at/), with substitution rates for A–G and C–G at 3.0351, and A–G and C–T at 6.7971. The consensus tree was generated via MrBayes version 3.2.7. Sequences from temperate regions are highlighted with a blue background, whereas those from tropical regions are highlighted with an orange background. The sequences from this study are indicated in bold. Trees were generated with 100 million iterations and a 25% burn-in rate. *Cytauxzoon felis* (KC207821) was determined as the outgroup

**Fig. 5:**
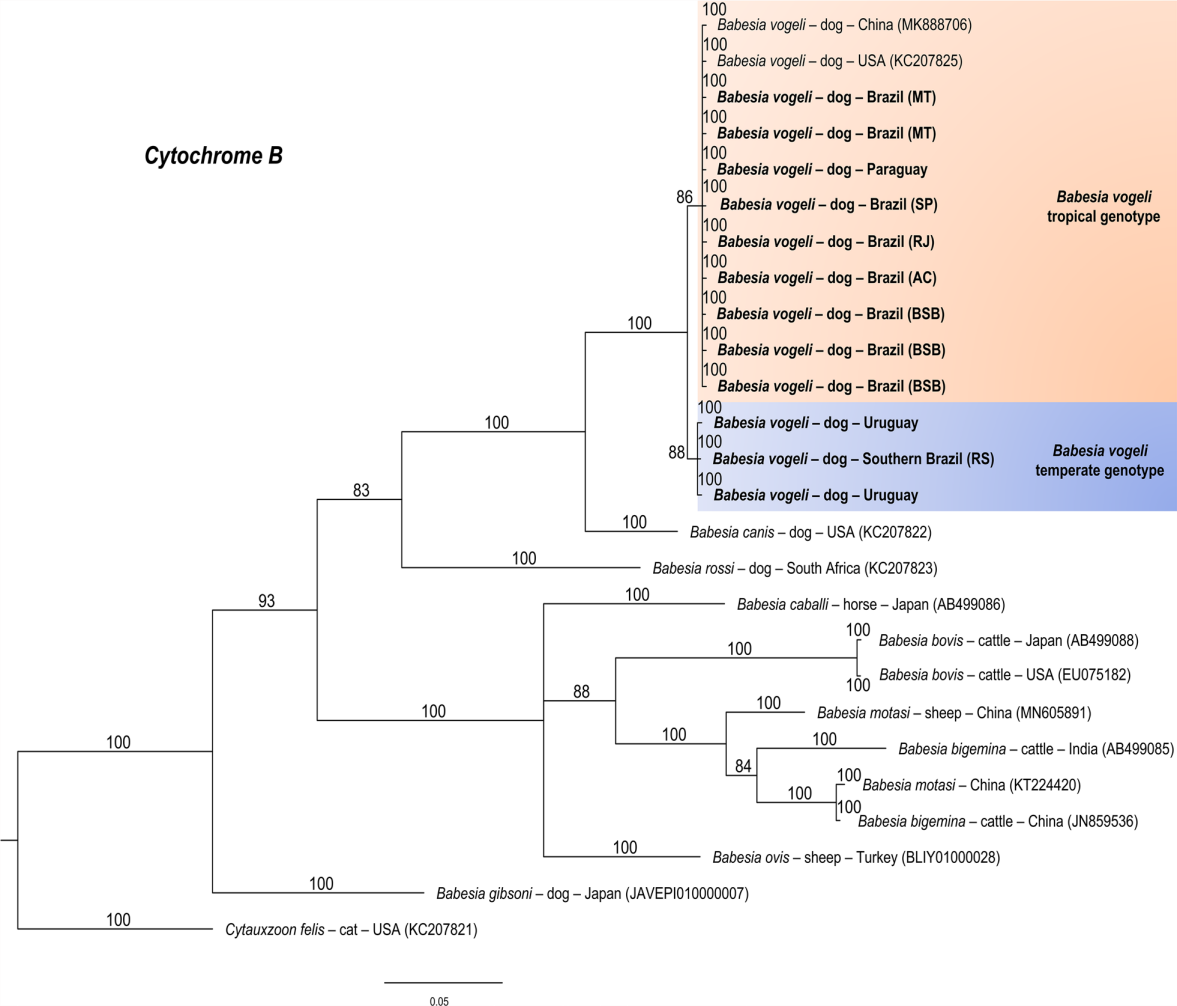
Bayesian phylogenetic tree based on the *cytb* gene constructed from a final alignment of 430 informative sites from nine sequences from this study and 14 homologous sequences. The substitution model (K3Pu + F + I + G4) was determined via the IQ-Tree web server (http://iqtree.cibiv.univie.ac.at/), with rate parameters for A–G and C–T of 8.0097 and 3.2377 for C–G and A–T (3.9615) (invariable sites: 0.1862). The consensus tree was generated via MrBayes version 3.2.7. Sequences from temperate regions are highlighted with a blue background, whereas those from tropical regions are highlighted with an orange background. The sequences from this study are indicated in bold. Trees were generated with 100 million iterations and a 25% burn-in rate. *Cytauxzoon felis* (KC207812) was used as an outgroup

For the *cox1*, approximately 900 base pairs (bp) were obtained for 9 of the 13 sequences. However, to increase the number of taxa in the analyses, only 652 bp were used, as most *B. vogeli* sequences in GenBank were up to 500 bp in length. Tests were performed for trees from 900 to 400 informative sites, yielding similar results, with no variation in branching patterns (Fig. 3). The *cox3* sequences yielded 641 informative sites in the final alignment, which also resulted in the separation of the two genotypes (Fig. 4). Although the *cytb* sequences reached ~900 bp, the phylogenetic tree based on 430 bp was able to discriminate the two genotypes (Fig. 5).

**Fig. 6:**
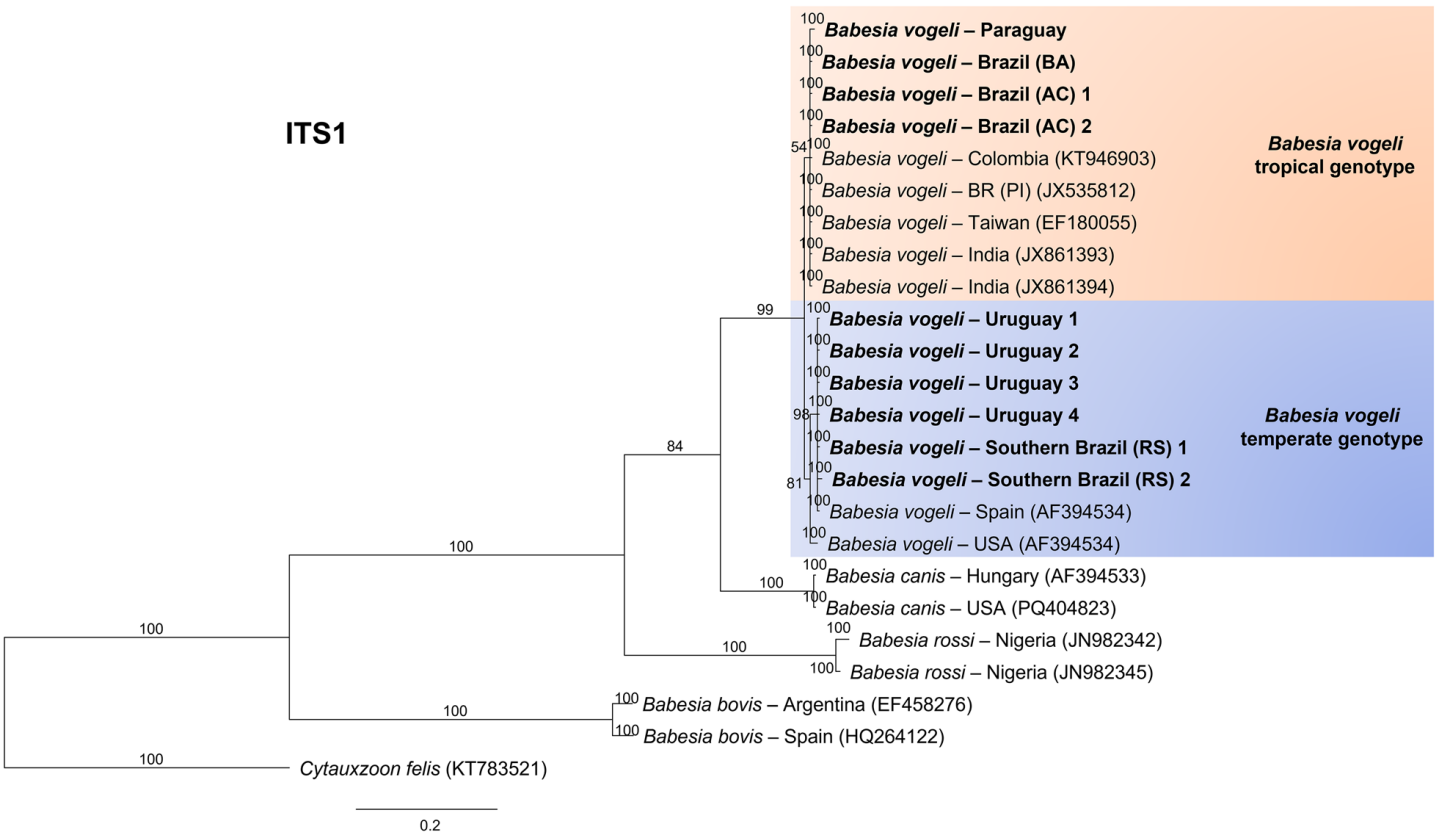
Bayesian phylogenetic tree based on intergenic transcribed spacer region 1 (ITS1) constructed from a final alignment of 324 informative sites, with 10 sequences from this study and 25 homologous sequences. The substitution model (K2P) was determined via the IQ-Tree web server (http://iqtree.cibiv.univie.ac.at/), with rate parameters for A–T (1.9487) and C–T (3.7724) (invariable sites, 0.516; gamma, 0.3757). The consensus tree was generated via MrBayes version 3.2.7. Sequences from temperate regions are highlighted with a blue background, whereas those from tropical regions are highlighted with an orange background. The sequences from this study are indicated in bold. Trees were generated with 100 million iterations and a 25% burn-in rate. *Cytauxzoon felis* (KT783521) was used as an outgroup

**Fig. 7:**
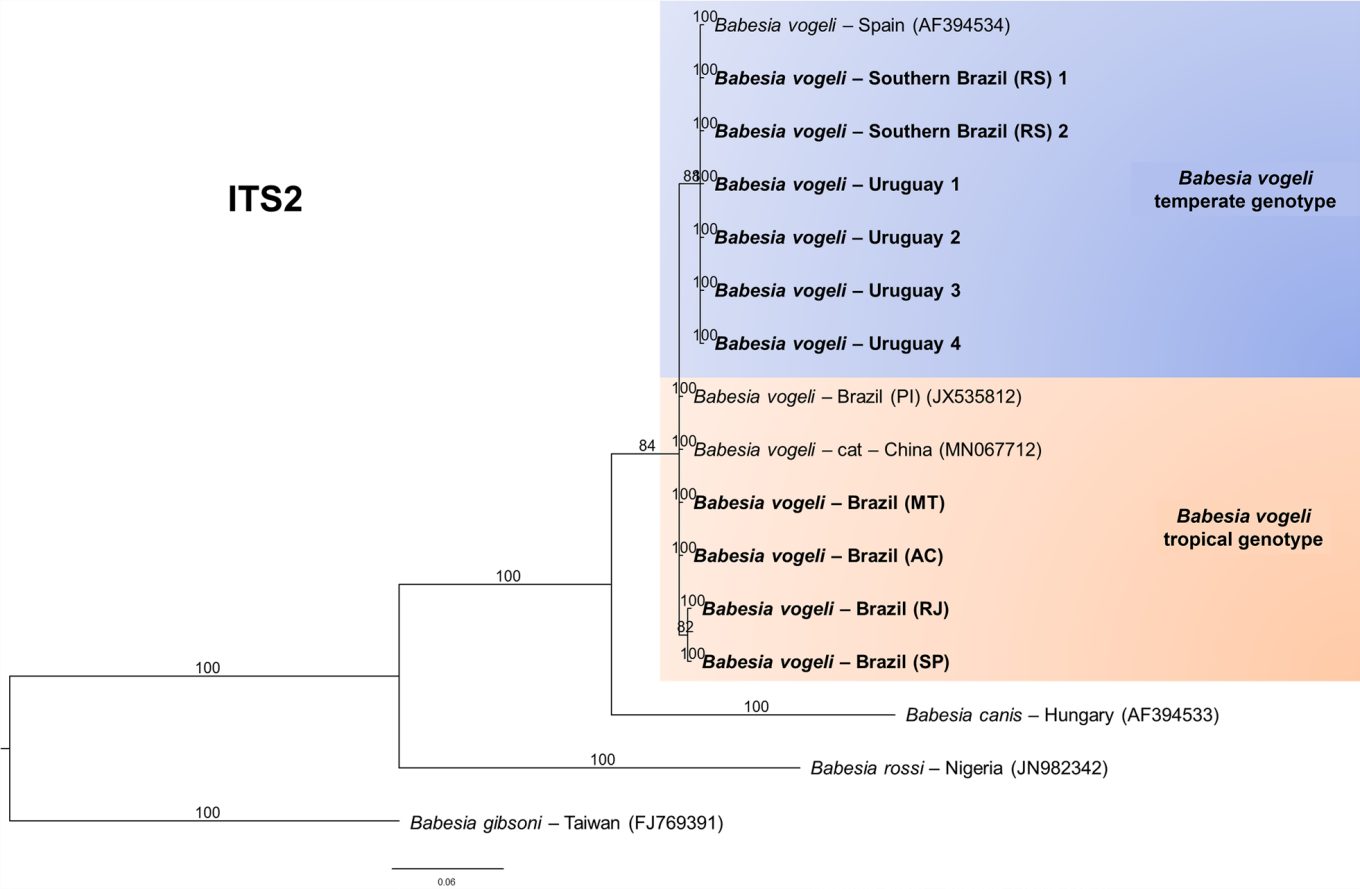
Bayesian phylogenetic tree based on the intergenic transcribed spacer region 2 (ITS2) constructed from a final alignment of 384 informative sites, with ten sequences from this study and six homologous sequences. The substitution model was determined via the IQ-Tree web server (http://iqtree.cibiv.univie.ac.at/), with rate parameters of A–G (2.8490) and C–T (2.8490) (invariable sites: 0.2958). The consensus tree was generated via MrBayes version 3.2.7. Sequences from temperate regions are highlighted with a blue background, whereas those from tropical regions are highlighted with an orange background. The sequences from this study are indicated in bold. Trees were generated with 100 million iterations and a 25% burn-in rate. *Babesia gibsoni* (FJ769391) was used as an outgroup

**Fig. 8:**
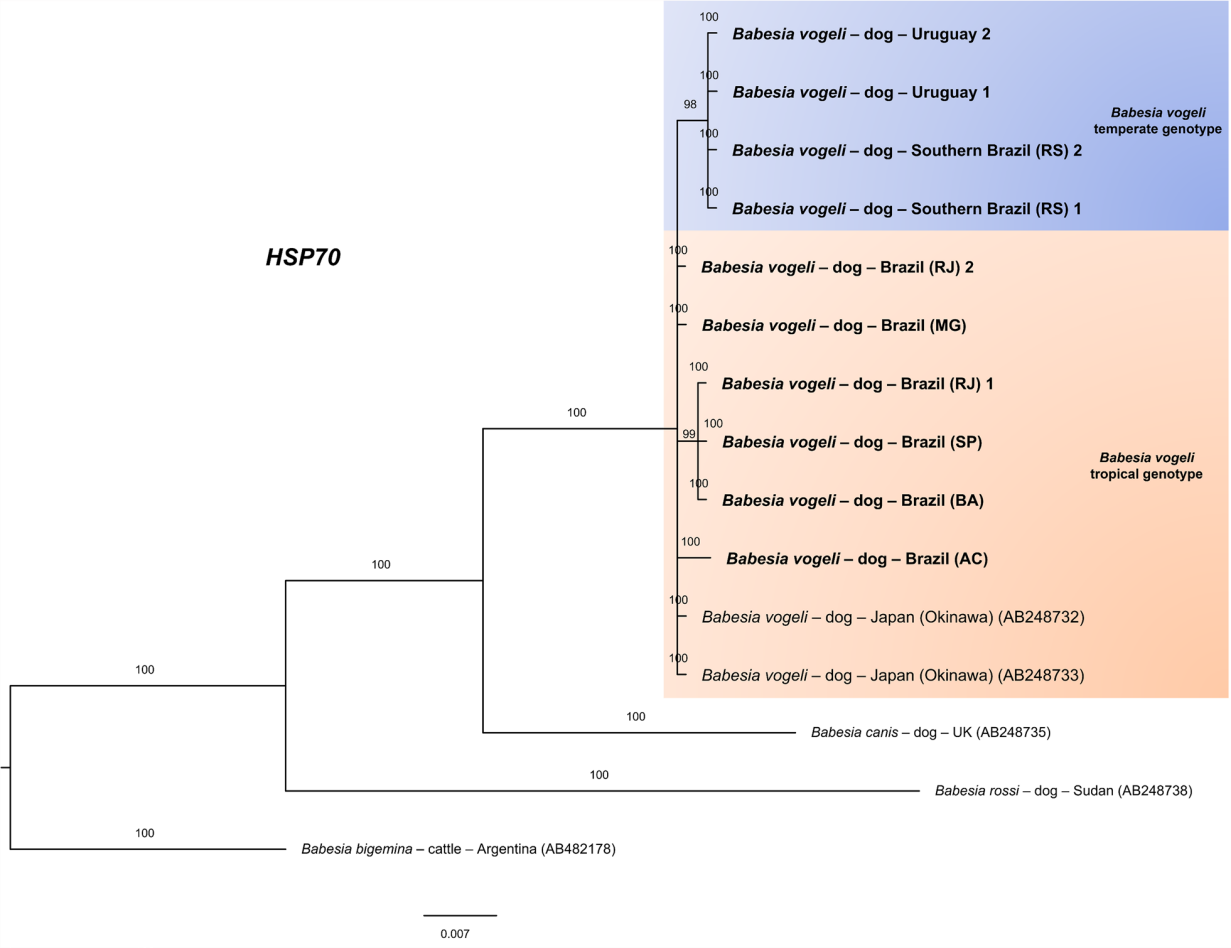
Bayesian phylogenetic tree based on heat shock protein 70 (*hsp70*) constructed from a final alignment of 840 informative sites, with ten sequences from this study and five homologous sequences retrieved from GenBank. The substitution model (TN + F + G4) was determined via the IQ-Tree web server (http://iqtree.cibiv.univie.ac.at/), with rate parameters of A–G (1.7379) and C–T (8.0580). The consensus tree was generated via MrBayes version 3.2.7. Sequences from temperate regions are highlighted with a blue background, whereas those from tropical regions are highlighted with an orange background. The sequences from this study are indicated in bold. Trees were generated with 100 million iterations and a 25% burn-in rate. *Babesia bigemina* (AB482178) was used as an outgroup

The results were similar for the ITS1 (Fig. 6), ITS2 (Fig. 7), and *hsp70* (Fig. 8), which yielded 324, 384, and 840 informative sites, respectively. The trees separated the genotypes according to climate zone.

**Fig. 9:**
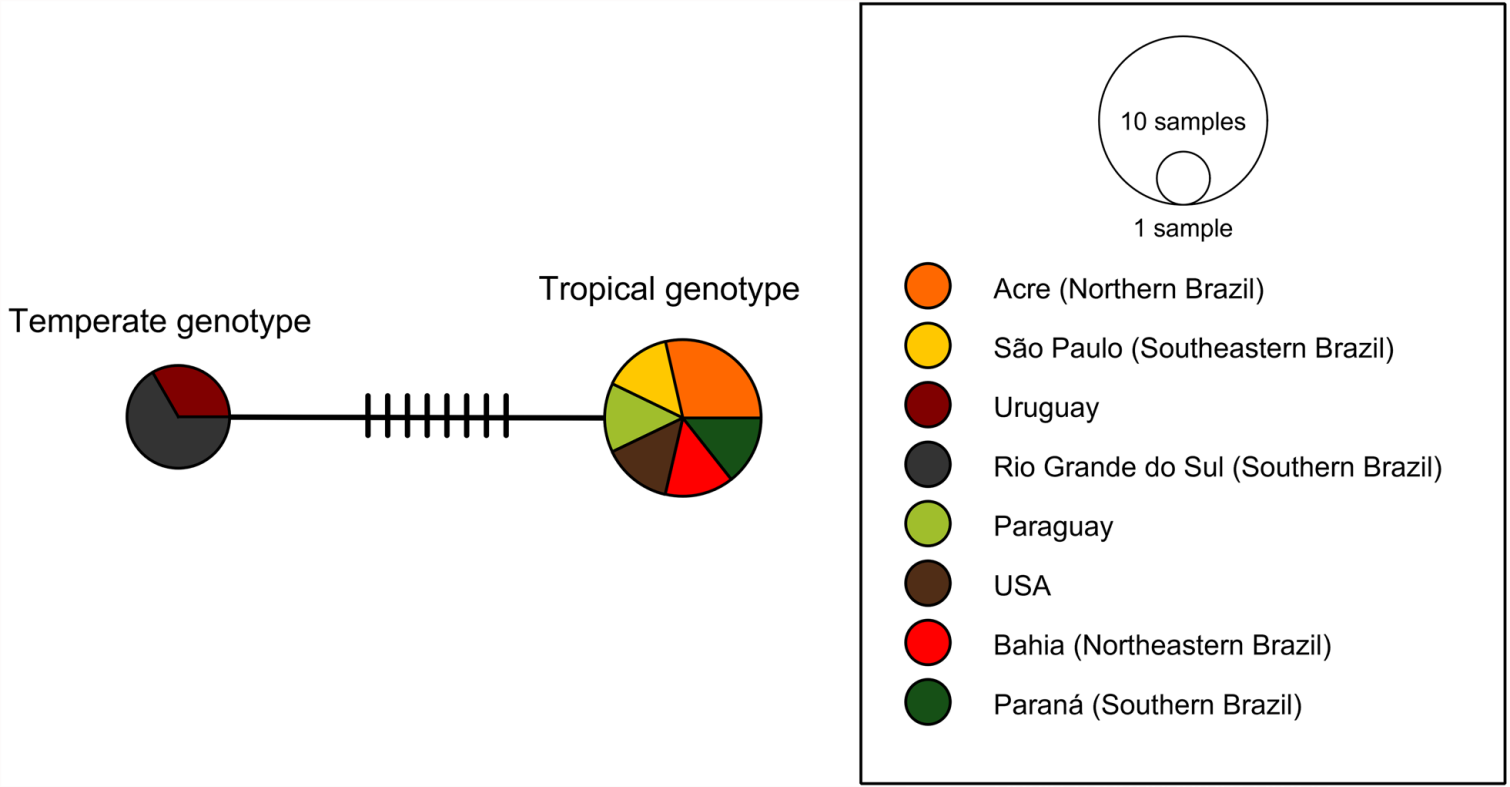
Haplotype network of mitochondrial cytochrome c oxidase 1 (*cox1*) gene sequences. The network depicts genetic variation among ten sequences (650 bp alignment length) derived from *B. vogeli* across temperate and tropical regions. Two divergent haplotypes were resolved, differing in eight mutational events. The population analysis was generated in PopArt version 1.7 via the TCS algorithm

**Fig. 10:**
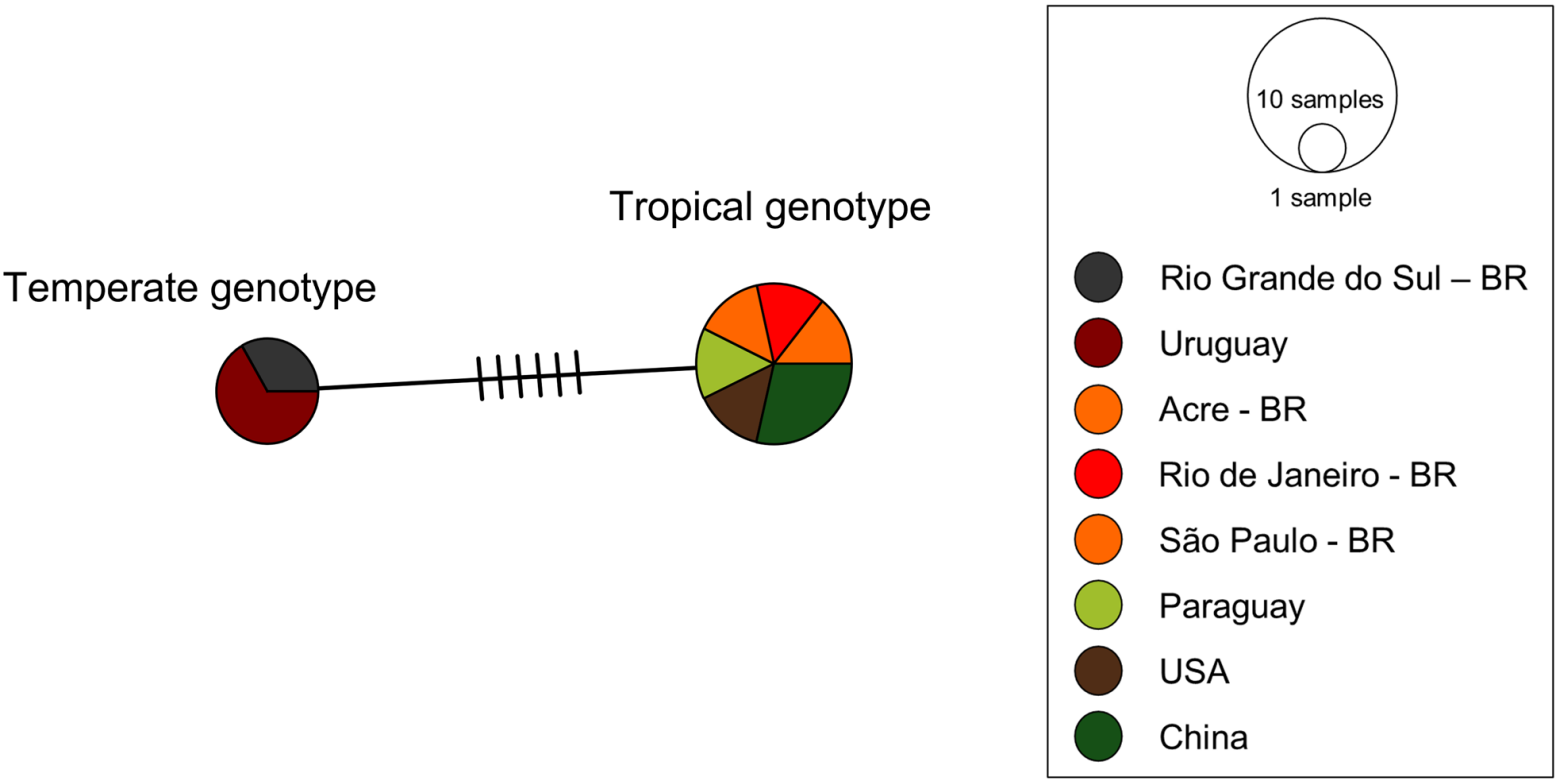
Haplotype network of mitochondrial cytochrome b (*cytb*) gene sequences. The network depicts genetic variation among ten sequences (625 bp alignment length) derived from *B. vogeli* across temperate and tropical regions. Two divergent haplotypes were resolved, differing in five mutational events. The population analysis was generated in PopArt version 1.7 via the TCS algorithm

**Fig. 11:**
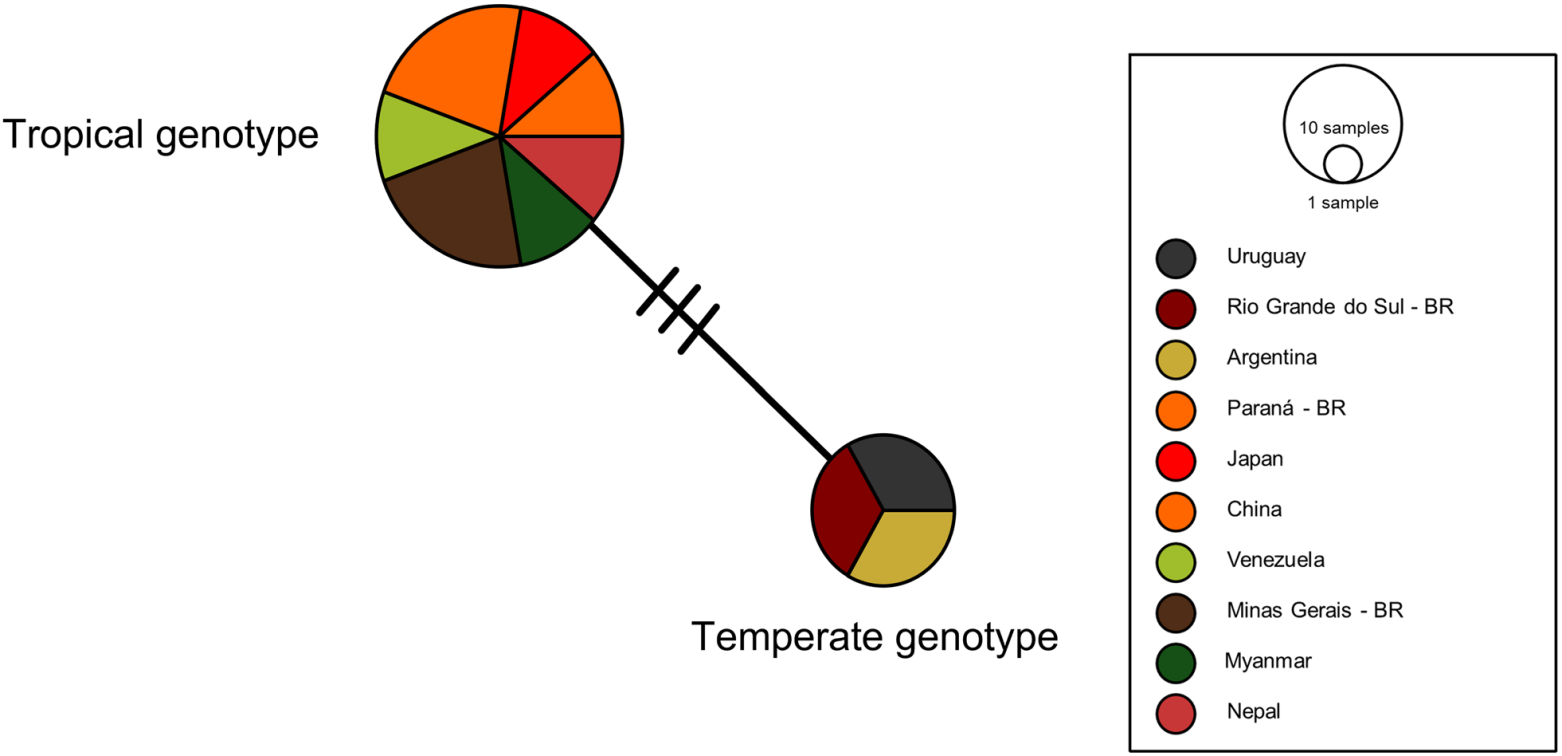
Haplotype network of 18S rRNA gene sequences. The network illustrates the genetic relationships among 12 sequences (1253 bp alignment length) derived from *B. vogeli* from temperate and tropical regions. Two distinct haplotypes were identified, separated by three mutational events. The population analysis was generated in PopArt version 1.7 via the TCS algorithm

Sequences from this study and GenBank were aligned for haplotype analysis; in addition, sequences of short length and short overlapping regions were removed. When 12 *cox1* sequences were aligned (10 from this study and 2 from GenBank), two haplotypes for *B. vogeli* were identified, with eight mutational events separating the temperate from the tropical genotype. Haplotype analysis for the *cytb* (*n* = 10 sequences—7 from this study and 3 from GenBank) and 18S rRNA (*n* = 12 sequences—3 from this study and 9 from GenBank) genes revealed five and three mutational events, respectively, between the tropical and temperate genotypes of *B. vogeli* (Figs. 9, 10, and 11). The results of haplotype analyses revealed two main haplotypes across all studied loci (*cox1*, 18S rRNA, and *cytb*). When compared with 18S rRNA, mitochondrial genes presented higher nucleotide diversity values (*Pi* = 0.00575 for *cox1* and 0.00449 for *cytb*) (*Pi* = 0.00098).

Of all samples tested (252), the screening primers (Bvog751F and Bvog930R) described in this study amplified the expected fragment size of the *hsp-70* gene from *B. vogeli*-positive samples, visible as a single band in agarose gel electrophoresis. Samples positive for *R. vitalii* and other pathogens tested in the same reaction were negative, resulting in an analytical specificity of 100%. In addition, the diluted samples used for testing their sensitivity were positive at all dilutions (30, 3, and 1 ng/μl).

## Discussion

Our findings demonstrate that *B. vogeli* populations exhibit geographic structuring, likely driven by the specificity of their respective vectors, *R. linnaei* and *R. sanguineus*, in tropical and temperate areas [7–9]. Haplotype analyses and phylogenetic inferences indicate that this divergence could reflect incipient speciation or the emergence of distinct genotypes. While the current observed genetic variations are limited to propose them as separate species, these populations likely represent two genetically distinct lineages, underscoring the role of vector adaptation in shaping *B. vogeli* microevolution.

BLAST analyses revealed that the 18S rRNA sequences of *B. vogeli* and *B. canis* are 99.0% similar, whereas the temperate and tropical genotypes of *B. vogeli* analyzed here are 99.8% identical. This high conservation in the 18S rRNA gene is consistent with patterns observed between distinct species, such as *B. capreoli* and *B. divergens* (99.8% similarity, differing by only three nucleotides [17]), which also share the same vector, *Ixodes ricinus* [17, 18], or *Theileria haneyi* and *T. equi* (99.9% similarity [19]). At the same time, the *B. vogeli* genotypes showed 99.5–99.8% similarity on the basis of the same gene. Mitochondrial markers such as *cox1* and *cytb* have proven critical in resolving this cryptic diversity. For example, Panait et al. [20] used mitochondrial loci to identify three novel *Cytauxzoon* spp. unresolved with 18S rRNA alone. Similarly, the temperate and tropical genotypes of *B. vogeli* in this study presented subtle but consistent differences in mitochondrial genes (*cox1* and *cytb*), supporting their classification as distinct genotypes rather than separate species. These findings align with the growing evidence that the 18S rRNA gene often obscures intraspecific genetic structuring [21], particularly in vector-borne parasites, for which host or vector specificity drives microevolution.

At the time of this study, 46 *B. vogeli* 18S rRNA sequences exceeding 1000 bp were available in GenBank, many of which originated from a single research group or geographic region. This limited the availability of sequences from distinct geographical areas, thereby reducing the robustness of the analysis. Shorter 18S rRNA sequences produced inconsistent branching patterns, even when supported by high statistical values, a concern also highlighted by another study [22], as short alignments may exclude variable regions where these are dissimilar. While limited data are available, the presence of temperate and tropical genotypes of *B. vogeli* in Europe and Africa, respectively, has been indicated, where *R. sanguineus* and *R. linnaei* are also found.

Although some ixodid tick species can transmit multiple *Babesia* spp., the reverse does not occur. *Babesia* spp. exhibit greater specificity toward their tick vectors [23], likely reflecting a long history of parasite–vector coevolution. This specificity may explain the genetic variation observed between the temperate and tropical genotypes of *B. vogeli* described in this study. To date, the vector competence of other ixodid tick species for *B. vogeli* remains unconfirmed, and no studies have tested whether tropical *B. vogeli* can be transmitted by *R. sanguineus* or temperate *B. vogeli* by *R. linnaei* [23]. A study in Tunisia revealed that only adult *R. sanguineus* s.l. could transmit *B. vogeli* from experimentally infected to naïve dogs [24]. In contrast, another study in Rio de Janeiro showed that both nymphs and adults of *R. linnaei* successfully transmitted *B. vogeli* to dogs [25].

Despite having different vectors in temperate and tropical regions, *B. vogeli* does not seem to cause different disease severity, suggesting coevolution between *B. vogeli* genotypes and domestic dogs. This could explain the relatively low pathogenicity of *B. vogeli* in dogs compared with other species such as *B. canis* and *B. rossi* [26]. While canine babesiosis appears to be less prevalent in temperate than in tropical regions of South America [27–29], further research is needed to compare clinical and hematological data and investigate possible differences in genotype virulence. In this regard, the transition from tropical to temperate climate in Brazil occurs in Santa Catarina, southern Brazil. While we received 30 samples from suspected canine piroplasmosis cases from this state, these cases were subsequently confirmed as *R. vitalii*, not *B. vogeli*.

Few studies included in a recent review on dog-associated *Babesia* spp. reported clinical signs suggesting a generally mild pathogenicity [6]. However, a direct comparison between tropical and temperate regions is needed to confirm this observation. The differentiation of *B. canis*, *B. vogeli*, and *B. rossi* into distinct species on the basis of invertebrate host specificity, pathogenicity, cross-immunity, and phylogenetic analyses [30, 31] suggests that a similar approach may be warranted for classifying the *B. vogeli* genotypes. Further studies investigating both vector lineages are needed, as has been carried out with *E. canis*, in which dogs infested with nymphs and adults of experimentally infected *R. linnaei* from São Paulo (Brazil) exhibited clinical signs, seroconversion, and PCR positivity for *E. canis*. In contrast, dogs infested with *R. sanguineus* (from Rio Grande do Sul [Brazil], Argentina, and Uruguay) previously fed on *E. canis*-experimentally infected dogs showed no clinical, serological, or PCR positivity [10]. Similar to *E. canis*, *B. vogeli* has a low prevalence rate in temperate regions, ranging from 0% (PCR) [28] to only 4% (PCR) in Rio Grande do Sul [27]. Despite reporting the seroprevalence of *B. vogeli*, the authors of this latter study suspected cross-reactivity with *R. vitalii*. In Argentina, prevalences ranging from 0.2% (cytology) to 3% (PCR) have been reported in the provinces of Buenos Aires, Córdoba, and Santa Fe [32, 33], where *R. sanguineus* is considered a possible vector [34]. This low prevalence was also observed in the present study, where most samples initially suspected of *B. vogeli* infection were found to be positive for other species, such as *R. vitalii* (3/252).

In contrast, in Brazilian regions where the vector is *R. linnaei*, molecular prevalence ranges from 2.5% in Mato Grosso state [35] to 9.7% in Brasília [36] and 15.7% in Rio de Janeiro [37]. Seroprevalence studies, however, report higher values: 57.9–83% in Pernambuco [29, 38] and 30.7–81.2% in Minas Gerais [29, 39]. In addition, when studying the bacterial microbiome of both tick species, researchers observed potential differences in *Coxiella* species between *R. sanguineus* and *R. linnaei*, suggesting that genetic differentiation within the *R. sanguineus* species complex might have driven the coevolution of distinct blood parasites [40].

The drivers of piroplasm speciation remain poorly understood, but vector diversity likely plays a role. For example, *Babesia pantanalensis* and *R. vitalii* are genetically close piroplasms (18S rRNA, 4.2% divergence; *cox1*, 4.9%) that infect the same host species and occupy regions with distinct vector populations [41, 42]. A similar situation is observed in temperate and tropical genotypes of *B. vogeli*, although their lower divergence may reflect a shorter evolutionary timescale.

Despite evidence from a study on mitochondrial genes of piroplasmids demonstrating the utility of mitochondrial genes for piroplasmid phylogenetic analyses [43], reliance on the 18S rRNA gene remains prevalent. A study has shown that the *cox1* gene exhibits high interspecific variation and low intraspecific variation, making it well-suited for distinguishing between genera and species but not for distinguishing between genotypes within a species [44]. Current GenBank records for the *cox1* and *cox3* gene sequences face limitations similar to those observed with 18S rRNA data, where available sequences are predominantly from India and Brazil, restricting broader phylogenetic analyses. For the *cytb* gene, only 42 sequences are currently deposited, with further constraints, as only seven exceed 1000 bp in length. Among these, one lacks collection metadata, whereas the remaining six originate exclusively from China. The limited availability of sequences for these molecular markers would have significantly limited the reliability of this study without access to geographically diverse samples and high-quality sequences. Notably, the *cox1* gene showed the most significant divergence (1.3%) among the genotypes described here. In contrast, *cytb* exhibited 0.9% divergence between genotypes, values similar to or greater than intraspecific levels for *Cytauxzoon* species, which ranged from 0% to 0.8% [45].

The haplotype analyses in this study revealed higher nucleotide diversity in mitochondrial genes (*cox1*, 0.00575; *cytb*, 0.00449) than in the 18S rRNA gene (0.00098), which is consistent with the faster evolutionary rate of mitochondrial loci in piroplasmids [20]. However, these values remain relatively low, supporting the classification of temperate and tropical *B. vogeli* as distinct genotypes rather than separate species. For example, the 18S rRNA divergence between the two genotypes (0.2%) falls well below the thresholds typically observed for species-level differentiation in *Babesia* spp. (e.g., 1.6–2.4% for *B. canis* versus *B. vogeli*) but is similar to or greater than the similarities between *B. capreoli* and *B. divergens* (0.2%), and *T. haneyi* and *T. equi* (0.1%).

Studies on the relationships and vector competence of different tick species are necessary to determine whether the proposed genotypes should be reclassified as genotypes, subspecies, or distinct species. Although the 18S rRNA gene is widely used for taxonomic purposes, standardizing the submission of longer *B. vogeli* sequences from both temperate and tropical regions to genetic databases seems necessary to improve the resolution of piroplasm phylogenetic trees, as well as for the other molecular markers mentioned here. Whole-genome sequencing of both genotypes of *B. vogeli* and closely related piroplasmids is greatly needed to shed light on the diversity of these parasites and their relationships with different tick species belonging to the *R. sanguineus* group worldwide [46].

## Conclusions

This study produced and analyzed *B. vogeli* sequences from regions where different tick species (*R. sanguineus* and *R. linnaei*) occur and identified two distinct genotypes in temperate and tropical regions. We suggest the nomenclature of “temperate” and “tropical” genotypes of *B. vogeli* for the respective regions. These terms could be used until a definitive taxonomic designation is eventually proposed for these genotypes.

## Supplementary Information

Additional File 1: Figure S1: Percent identity between 18S rRNA sequences of *Babesia vogeli* from this study (bold) and sequences of *B. vogeli*, *Babesia canis*, *Babesia gibsoni*, and *Babesia rossi* from GenBank. Figure S2. Percent identity between *hsp70* sequences from *Babesia vogeli* from this study (bold) and sequences from *B. vogeli*,* Babesia canis*, and *Babesia rossi* from GenBank. Figure S3. Percent identity between ITS1 sequences from *Babesia vogeli* from this study (bold) and sequences from *B. vogeli*, *Babesia canis*, and *Babesia rossi* from GenBank. Figure S4. Percent identity between ITS2 sequences from *Babesia vogeli* from this study (bold) and sequences from *B. vogeli*, *Babesia canis*, and *Babesia rossi* from GenBank. Figure S5. Percent identity between *cox1* sequences from *Babesia vogeli* from this study and sequences of *B. vogeli* and *Babesia canis* from GenBank. Figure S6. Percent identity between *cox3* sequences from *Babesia vogeli* from this study (bold) and sequences from *B. vogeli* and *Babesia canis* from GenBank.

## Data Availability

The data supporting this article’s main conclusions are included in the article and its Additional File 1. Sequences generated in this study have been deposited in GenBank: 18 s rRNA (PV546615, PV546616, PV546617, PV546618, PV546619, PV546620, PV546621); ITS (PV548080, PV548081, PV548082, PV548083, PV548084, PV548085, PV548086, PV548087, PV548088, PV548089, PV548090, PV548091, PV548092, PV548093); cox1 (PV565252, PV565253, PV565254, PV565255, PV565256, PV565257, PV565258, PV565259, PV565260, PV565261, PV565262, PV565263); cox3 (PV565264, PV565265, PV565266, PV565267, PV565268, PV565269, PV565270, PV565271, PV565272, PV565273, PV565274, PV565275, PV565276, PV565277, PV565278, PV565279, PV565280); cytb (PV565281, PV565282, PV565283, PV565284, PV565285, PV565286, PV565287, PV565288, PV565289, PV565290, PV565291, PV565292, PV565293, PV565294, PV565295); hsp70 (PV565296, PV565297, PV565298, PV565299, PV565300, PV565301, PV565302, PV565303, PV565304, PV565305, PV565306).
