# Supplementary material for: Are Babesia vogeli genotypes associated with Rhipicephalus sanguineus and Rhipicephalus linnaei distribution?
Source: Parasit Vectors. 2025 Jul 13;18:282. doi: 10.1186/s13071-025-06923-8 (PMC12257672; doi:10.1186/s13071-025-06923-8)
Supplement: Supplementary file 1 — Additional File 1: Figure S1: Percent identity between 18S rRNA sequences of Babesia vogeli from this study (bold) and sequences of B. vogeli, Babesia canis, Babesia gibsoni, and Babesia rossi from GenBank. Figure S2. Percent identity between hsp70 sequences from Babesia vogeli from this study (bold) and sequences from B. vogeli, Babesia canis, and Babesia rossi from GenBank. Figure S3. Percent identity between ITS1 sequences from Babesia vogeli from this study (bold) and sequences from B. vogeli, Babesia canis, and Babesia rossi from GenBank. Figure S4. Percent identity between ITS2 sequences from Babesia vogeli from this study (bold) and sequences from B. vogeli, Babesia canis, and Babesia rossi from GenBank. Figure S5. Percent identity between cox1 sequences from Babesia vogeli from this study and sequences of B. vogeli and Babesia canis from GenBank. Figure S6. Percent identity between cox3 sequences from Babesia vogeli from this study (bold) and sequences from B. vogeli and Babesia canis from GenBank. [file 13071_2025_6923_MOESM1_ESM.pptx]

## Slide 1
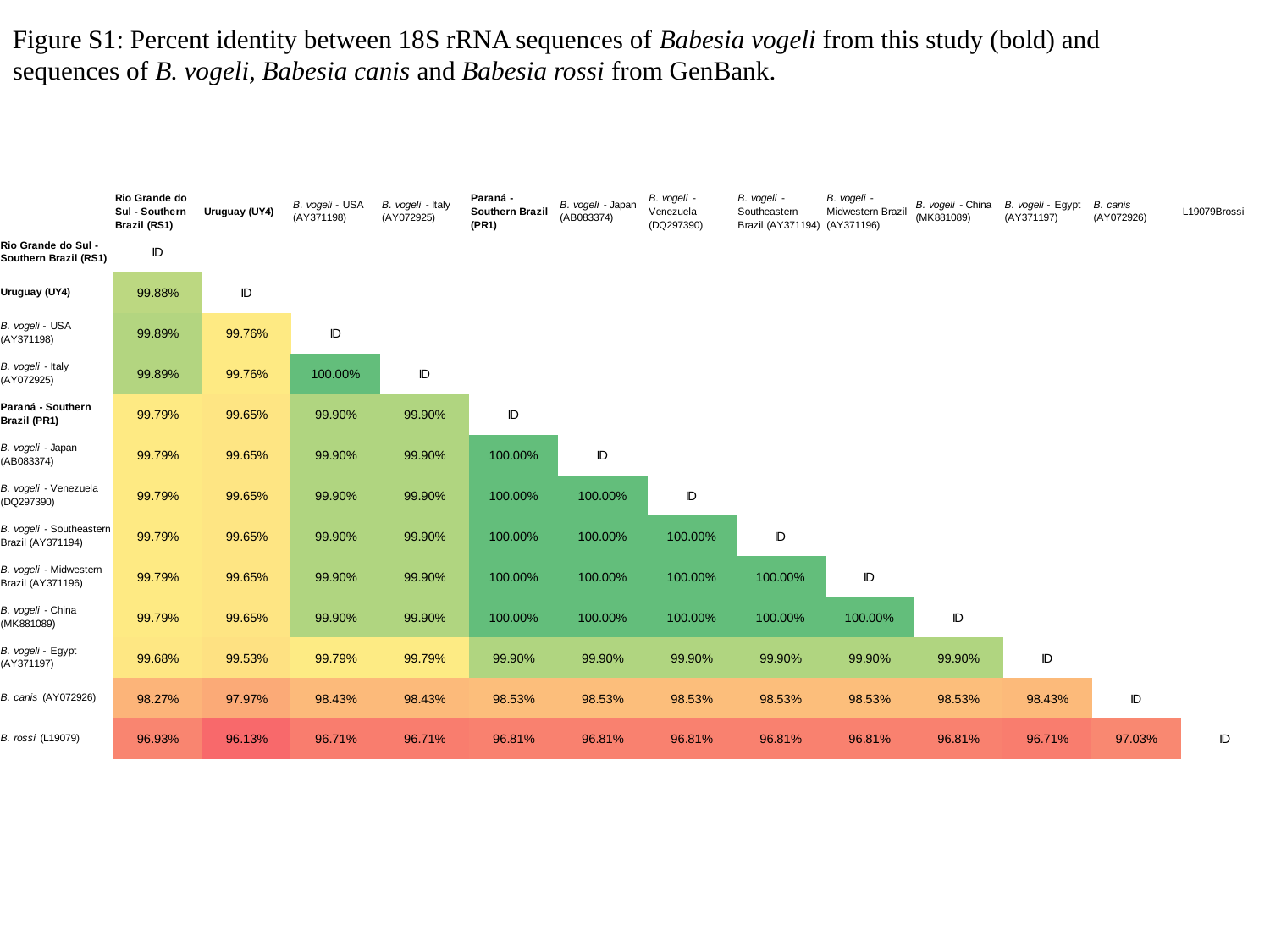

Figure S1: Percent identity between 18S rRNA sequences of Babesia vogeli from this study (bold) and sequences of B. vogeli, Babesia canis and Babesia rossi from GenBank.

## Slide 2
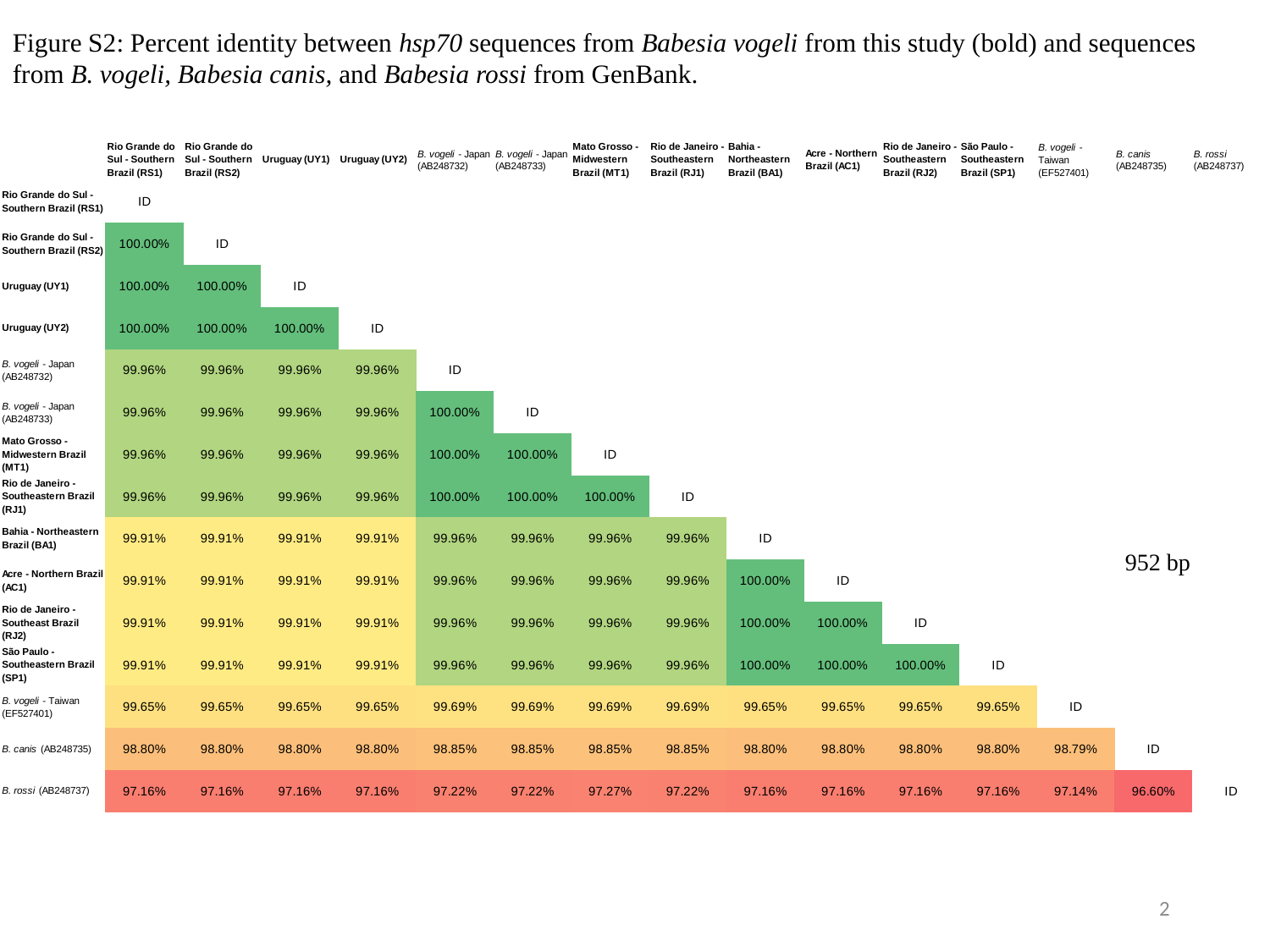

Figure S2: Percent identity between hsp70 sequences from Babesia vogeli from this study (bold) and sequences from B. vogeli, Babesia canis, and Babesia rossi from GenBank.
952 bp
2

## Slide 3
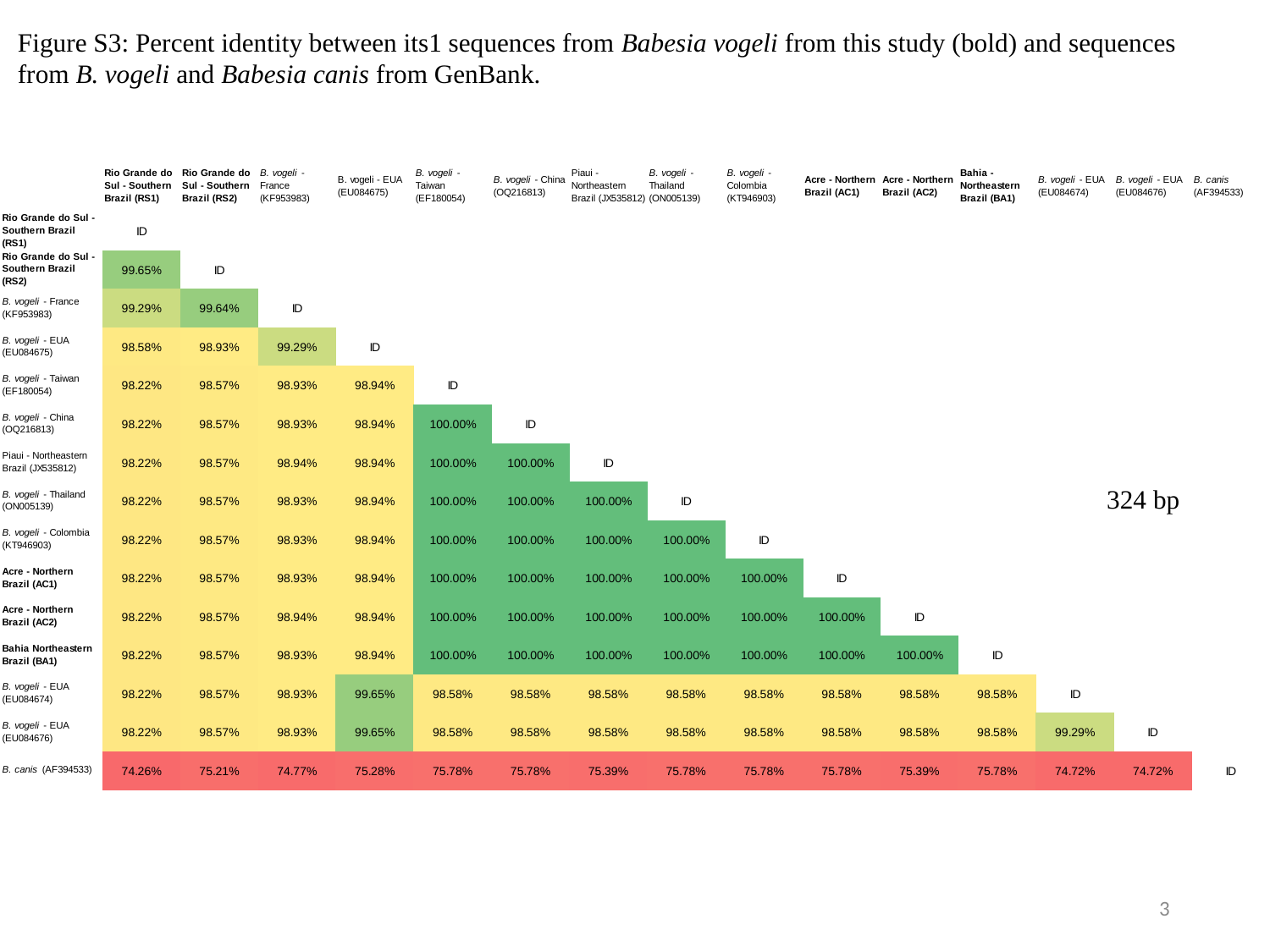

Figure S3: Percent identity between its1 sequences from Babesia vogeli from this study (bold) and sequences from B. vogeli and Babesia canis from GenBank.
324 bp
3

## Slide 4
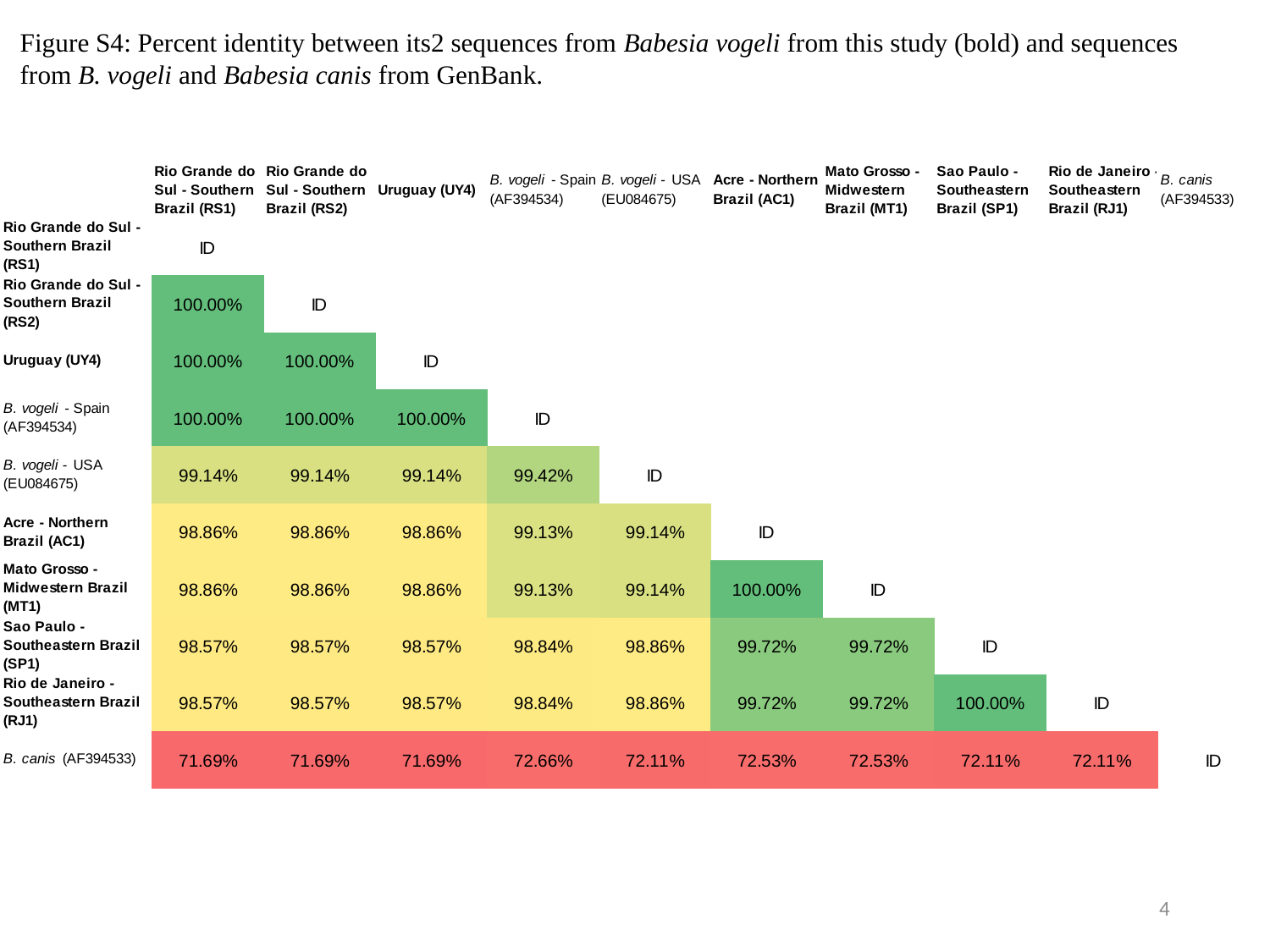

Figure S4: Percent identity between its2 sequences from Babesia vogeli from this study (bold) and sequences from B. vogeli and Babesia canis from GenBank.
4

## Slide 5
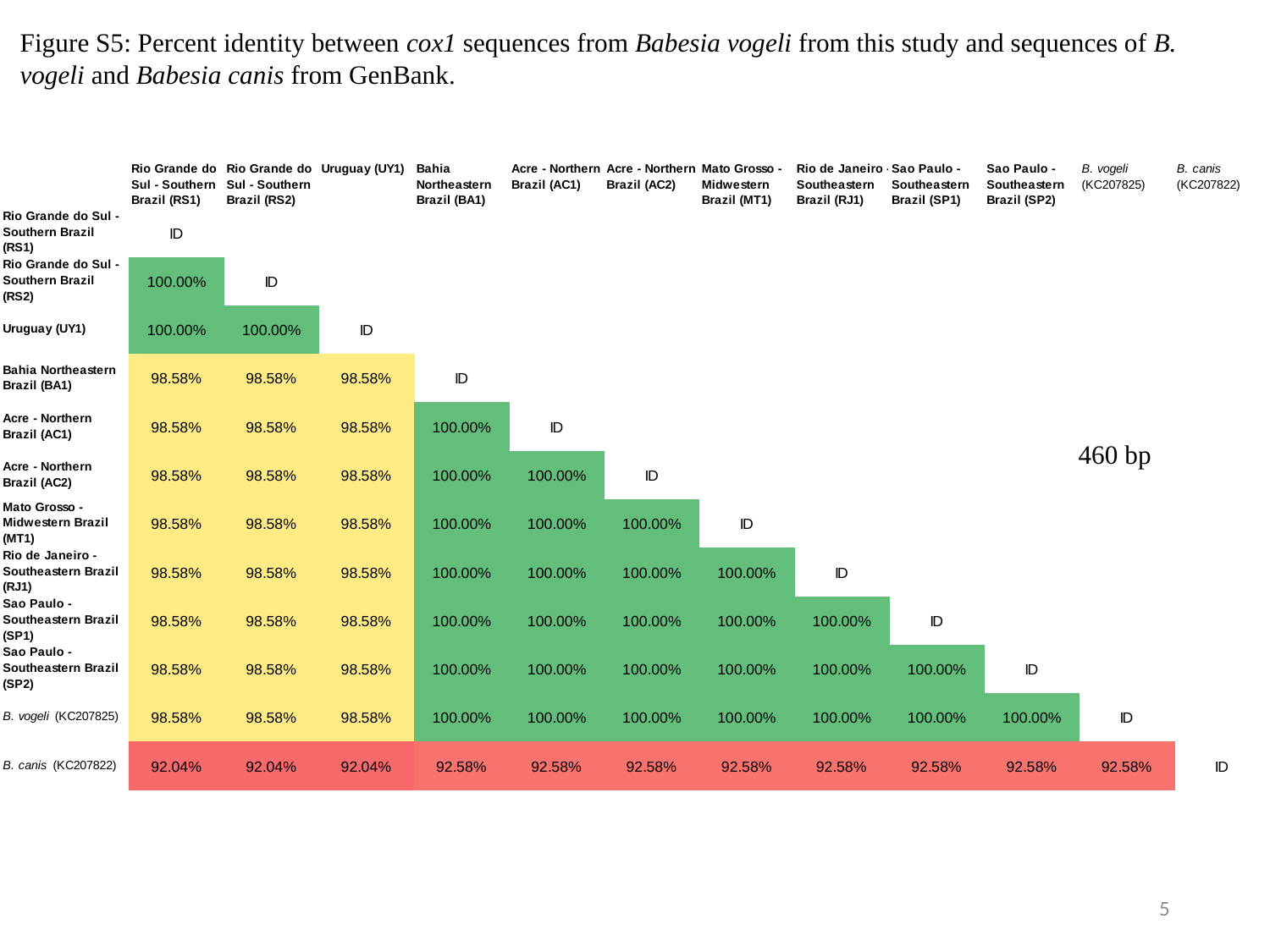

Figure S5: Percent identity between cox1 sequences from Babesia vogeli from this study and sequences of B. vogeli and Babesia canis from GenBank.
460 bp
5

## Slide 6
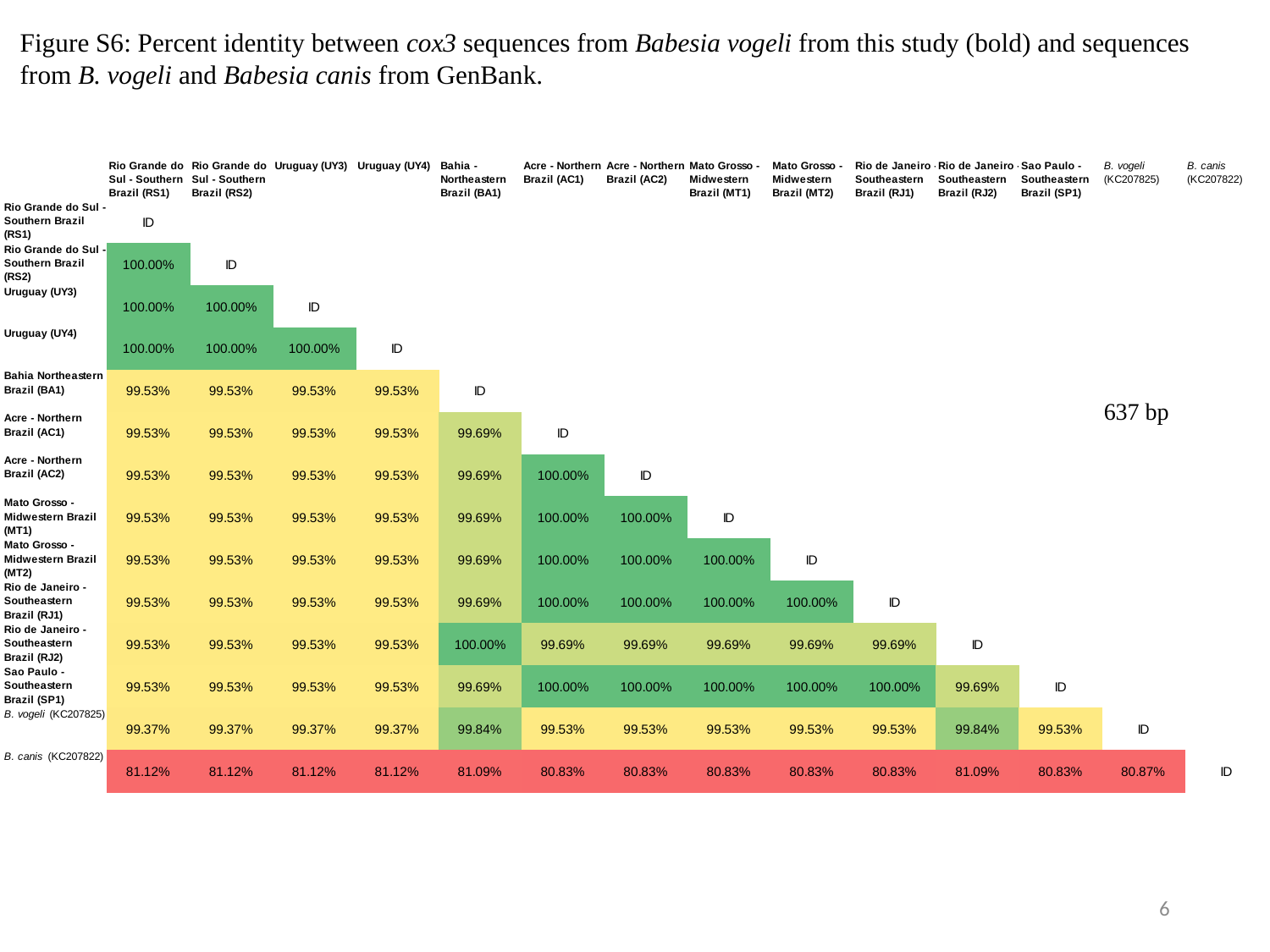

Figure S6: Percent identity between cox3 sequences from Babesia vogeli from this study (bold) and sequences from B. vogeli and Babesia canis from GenBank.
637 bp
6
